# Polymeric Membranes Incorporated With ZnO Nanoparticles for Membrane Fouling Mitigation: A Brief Review

**DOI:** 10.3389/fchem.2020.00224

**Published:** 2020-04-08

**Authors:** Liguo Shen, Zhengyi Huang, Ying Liu, Renjie Li, Yanchao Xu, Gjon Jakaj, Hongjun Lin

**Affiliations:** College of Geography and Environmental Sciences, Zhejiang Normal University, Jinhua, China

**Keywords:** polymeric membrane, modification, ZnO nanoparticles, composite membrane, antifouling

## Abstract

Due to the flexibility of operation, high removal ability, and economic cost, separation membranes have proved to be one of the most significant technologies in various aspects including water treatment. However, membrane fouling is a predominant barrier which is severely limiting the whole membrane industry. To mitigate membrane fouling, researchers have carried out several modification strategies including the incorporation of hydrophilic inorganic components. Zinc oxide (ZnO) nanoparticles, known as a low-cost, environment-friendly, and hydrophilic inorganic material, have been used by worldwide researchers. As claimed by the scientific literatures, ZnO nanoparticles can not only endow the polymeric membranes with antifouling performance but also supply a photocatalytic self-cleaning ability. Therefore, polymer–ZnO composite membranes were considered to be an attractive hot topic in membrane technology. In the last decades, it has been significantly matured by a large mass of literature reports. The current review highlights the latest findings in polymeric membranes incorporated with ZnO nanoparticles for membrane fouling mitigation. The membrane fouling, ZnO nanoparticles, and modification technology were introduced in the first three sections. Particularly, the review makes a summary of the reports of polyvinylidene fluoride (PVDF)–ZnO composite membranes, polyethersulfone (PES)–ZnO composite membranes, and other composite membranes incorporated with ZnO nanoparticles. This review further points out several crucial topics for the future development of polymer–ZnO composite membranes.

**Graphical Abstract:**
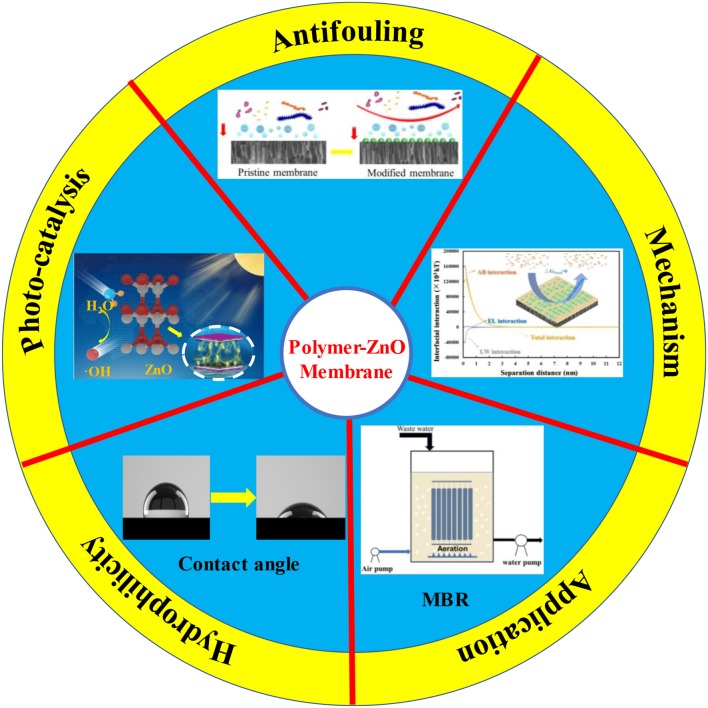
The study and applications of polymer-ZnO membranes.

## Introduction

Water resource is currently facing an extreme scarcity crisis due to poor management, overuse, environmental degradation, contamination, and booming of the population (Eliasson, 2015; Vörösmarty et al., 2015). In anticipation of the rising negative consequences, various water treatment technologies are being conducted in response to the urgent growing stresses (Chong et al., 2010; Pendergast and Hoek, 2011). Comparing to the traditional water treatment technologies, pressure-driven membrane technology is attracting worldwide attention due to its obvious superiorities (Strathmann, 1981; Baker, 2002; Padaki et al., 2015). Therefore, membrane separation technology has found extensive applications in the water treatment field (Ang et al., 2015; Subramani and Jacangelo, 2015; Tran et al., 2015).

Membrane can be defined as a physical interphase and acts as a selective barrier for different chemical species in a separation procedure. With a long period of development, membranes now appear in abundant categories, such as homogeneous or heterogeneous membranes; symmetric or asymmetric membranes (Lin et al., 2006); positively charged, negatively charged, or neutral membranes (Schaep and Vandecasteele, 2001). According to the membrane pore sizes, the pressure-driving membranes can beusually categorized into microfiltration membrane (MF), ultrafiltration membrane (UF), nanofiltration membrane (NF), and reverse osmosis membrane (RO) (Van Der Bruggen et al., 2003). Generally, MF, UF, NF, and RO are applied to effectively reject suspended particles, macromolecules, multivalent ions, and monovalent ions from the water system, respectively.

Particularly, membranes are significantly differentiated through the membrane materials, such as inorganic ceramic membranes and organic polymeric membranes (Anderson et al., 1988; Meng et al., 2009; Kim and Van der Bruggen, 2010; Lee et al., 2011; Kang and Cao, 2014). The organic polymers possess outstanding mechanical, physical, and chemical properties, therefore, become the widely used commercial materials. Recently, researchers have developed various polymeric membranes (Shao and Huang, 2007; Wang et al., 2013). Among the polymeric membranes, it is believed that polyethersulfone (PES) and polyvinylidene fluoride (PVDF) mainly occupy the membrane market. The inorganic membranes are usually made from ceramic materials such as Al_2_O_3_, SiO_2_, or TiO_2_ nanoparticles and so on (Mallada and Menéndez, 2008). Comparing to polymeric membranes, they cannot find extensive applications due to their exorbitant price and fragility which threatens the integrity of membranes. Therefore, polymeric membranes are attracting more and more worldwide attention.

However, the membrane fouling is seriously limiting the development of polymeric membranes due to their intrinsic hydrophobicity which favors to adhere the hydrophobic natural organic matters (NOMs) (Lin et al., 2013; She et al., 2016). In view of this, researchers make their efforts to hydrophilization modification *via* various methods (Zhao et al., 2013; Kang and Cao, 2014). Especially, the combination of polymer and nanoparticles was verified to be a successful strategy to enhance membrane hydrophilicity (Cuiming et al., 2003; Wu et al., 2003). Recently, this strategy is becoming more and more popular in antifouling research (Kango et al., 2013; Ng et al., 2013; Duan et al., 2015). Based on the abundant literatures, TiO_2_ nanocomposite-based polymeric membranes have already been well-studied and reviewed by previous reports (Kwak et al., 2001; Leong et al., 2014; Bet-Moushoul et al., 2016). Meanwhile, plenty of research articles have proved that zinc oxide (ZnO) nanoparticles, with one-fourth the cost of TiO_2_, presented comparable physical and chemical properties with TiO_2_ (Hong and He, 2014). Therefore, ZnO nanoparticles are believed to be a competitive alternative to TiO_2_ nanoparticles in the formation of antifouling organic–inorganic composite membranes (Balta et al., 2012). Although extensive articles have developed polymer–ZnO composite membranes for antifouling applications (Leo et al., 2012; Zhao S. et al., 2015), a comprehensive review of literature of polymer–ZnO composite membranes is still limited and highly desired in the membrane field.

This review will highlight the latest findings in polymeric membranes incorporated with ZnO nanoparticles for membrane fouling mitigation. The membrane fouling, ZnO nanoparticles, and modification technology were introduced in the first three sections. Particularly, the review introduced the reports of PVDF–ZnO composite membranes, PES–ZnO composite membranes, and other composite membranes incorporated with ZnO nanoparticles. The current study further points out several crucial topics for the future development of polymer–ZnO composite membranes.

## Membrane Fouling

Membrane fouling, the most troublesome problem for the whole membrane technology industry (Gao et al., 2011; Tijing et al., 2015), extremely cripples the membrane performance and drastically increases the operation costs. The membrane fouling resulted from a complicated interaction process between the membrane and the foulant during the filtration operation (Lin H. et al., 2014). The complicated interaction process is commonly involved with many characteristics, such as membrane pore/surface structure, pollutant conditions, charge property, and particularly the hydrophobicity (Chen et al., 2016; Teng et al., 2020; You et al., 2020). To simplify an extremely complicated process, researchers studied the membrane fouling from external fouling and internal fouling (Ma et al., 2001).

Membrane fouling usually occurs through the deposition of foulant sediments from wastewater onto the membrane surface, which results in a “cake layer,” known as external fouling. However, some small foulant particles go into the sublayer of the membrane and eventually adhere onto the pore walls during the filtration process. Moreover, the foulant materials at the porous support layer can further combine with the previously adsorbed particles. In this way, it will lead to “pore-clogging,” which is known as internal fouling. Comparing with the external fouling, the internal fouling is less reversible (Kimura et al., 2004; Shirasaki et al., 2008). The backwash method has been developed for the removal of the foulant within the support layer (Crozes et al., 1997; Smith et al., 2006; Hwang et al., 2009). It displays an efficient removal for the external fouling on the membrane surface but cannot efficiently remove the internal fouling. Recently, it is found that the surface modification can promote the hydrophilic ability of membrane and has displayed successfully applications in reducing the external fouling (Ma et al., 2001; Shen et al., 2017a). However, internal fouling control remains in a complicated situation and is still an attractive research topic for future studies.

To obtain a hydrodynamic understanding of the membrane fouling, an interpretation of the membrane fouling mechanism can be now developed from the extended Derjaguin–Landau–Verwey–Overbeek (XDLVO) theory (Lee et al., 2007; Hong et al., 2013; Lin T. et al., 2014). Based on the XDLVO theory, the fouling process can be quantitatively evaluated *via* the interface thermodynamic reactions (Shen et al., 2015, 2017b; Yu et al., 2019). Theoretically, a membrane that presents lower roughness, an enhanced negative charge, and a hydrophilic surface will show less adhesion force toward NOM present in wastewater (Zhang et al., 2015). Recently, researchers employed more methods to explain the membrane fouling mechanism such as density functional theory (DFT), computational fluid dynamics (CFD), artificial neural network (ANN), and combined methods (Ghidossi et al., 2006; Teng et al., 2018; Zhang et al., 2018; Chen et al., 2019; Li et al., 2019). These theoretical results not only give out a quantitative interpretation to membrane fouling process but also can direct the modification of membranes.

## Zinc Oxide Nanoparticles

ZnO, as an outstanding material (Look, 2001), has been widely used in photocatalysis and antifouling purposes (Wu and Xue, 2011). Moreover, the high surface-to-volume ratio of ZnO nanoparticles makes it a significant candidate in various fields (Ko et al., 2011). With one-fourth the cost of TiO_2_, ZnO nanoparticles have been even considered to be an important alternative to TiO_2_ (Hong and He, 2014). ZnO nanoparticles possess a strong hydrophilicity by adhering the hydrophilic functional groups such as –OH, –SO_3_H, and –COOH (Shen et al., 2012). Thanks to the outstanding properties of ZnO nanoparticles, an increasing number of researchers are able to promote the membrane's antifouling ability by combining ZnO nanoparticles with the polymeric membrane (Liang et al., 2012).

## Modification Technology

Since fouling happens at the surface and inside of the membranes, the modification strategies are generally classified into two types: external/surface modification and internal/bulk modification technologies.

For internal modification, the casting membrane solution is firstly mixed with a certain amount of ZnO nanoparticles, then transferred to a glass plate for the membrane preparation via the phase inversion method. By this method, the ZnO nanoparticles were embedded into the whole polymeric networks. Very small amounts of ZnO nanoparticles were observed at the membrane, which could be attributed to the solvent/non-solvent exchange process. This exchange process is an easy-going method without additional chemicals which are environmentally unfriendly and increasing cost. Therefore, this method has been employed by many researchers and reveals a great potential for further applications. For example, Ahmad et al. (2015) dispersed hydrophilic ZnO nanoparticles (0–3.75 wt.%) into PES casting solution to prepare a hydrophilic and antifouling composite membrane. Moreover, Hong and He (2012) modified PVDF membranes by the addition of 0–1 wt.% ZnO nanoparticles *via* the phase inversion method. Researchers have also tried to introduce ZnO nanoparticles into various types of polymeric materials, such as polyvinyl chloride (PVC) (Rabiee et al., 2015), polystyrene (PS) (Leo et al., 2012), polyethylene (PE) (Jafarzadeh et al., 2015), and so on. The internal modification reveals many advantages and delivers a reliable candidate method for preparing the ZnO incorporated polymeric membranes. However, some critical and intractable problems still need to be dealt with. One of the key problems is the aggregation of nanoparticles, which is the nature of all nanoparticles and greatly limits the filtration capacity of ZnO in the polymeric membranes (Liang et al., 2012). In addition, the leaking of ZnO nanoparticles is an additional problem that appears during the application of composite membranes. The ZnO nanoparticles are usually embedded into the polymeric network through a physical interaction that is not strong enough to fasten all the ZnO nanoparticles. As a result, nanoparticles are always discharged during the filtration process. Therefore, overcoming aggregation and leaking of ZnO nanoparticles in the polymeric membrane is a key research topic in the future of ZnO nanoparticle-modified polymeric membranes.

The external modification refers to physically or chemically coating hydrophilic components onto the membrane surface (Yu et al., 2019; Zhao et al., 2019). During the membrane fouling process, pollutants firstly get closer to the membrane surface, then interact with the membrane surface, and eventually adhere to the membrane surface. In view of this, it is claimed that the membrane surface is the critical functional layer which mainly determines the membrane fouling ability (Elimelech et al., 1997). Accordingly, modification of the membrane surface plays a more directive and more efficient role for membrane antifouling. Based on this concept, the surface-modified polymeric membranes by ZnO nanoparticles have been developed and show great promotion in antifouling ability. Since the ZnO nanoparticles are located on the membrane surface, their efficiency is largely enhanced during the antifouling process. Another prominent advantage is the possibility to chemically bond the ZnO nanoparticles on the membrane surface *via* bridging the chemical reaction between ZnO nanoparticles and the polymeric membrane. For example, Li et al. (2016) employed the atomic layer deposition (ALD) technique to modify the PVDF membrane surface. By this technique, an ultrathin ZnO layer can be efficiently formed on the membrane surface after 10 ALD cycles. Therefore, the membrane's hydrophilicity and separation performance were significantly enhanced. Unlike the internal modification, the surface modification method requires complicated experimental operations and additional chemicals that are not good to a competitive membrane cost. From the experimental results, it can be concluded that internal modification is an easier method to obtain enhanced antifouling membrane; however, the higher efficiency and the larger selectivity of grafting agents ensure the surface modification method to be the preferred adopted method. To obtain the synergetic advantages of external and internal modification, a novel strategy has been developed by using the external magnetic field which can fix the magnetic nanoparticles onto the membrane surface. Recently, this novel concept has been used to polymer–carbon nanotube membranes (Yu et al., 2020) and polymer–graphene oxide membranes (Xu et al., 2016). It is believed that it will have a potential application in polymer–ZnO membranes.

## Zinc Oxide-Incorporated Polymeric Membranes

### Polyvinylidene Fluoride Membrane Incorporated With Zinc Oxide Nanoparticles

PVDF has been extensively applied in separation technology for various purposes (Liu et al., 2011; Kang and Cao, 2014). However, the membrane fouling is shortening PVDF membranes' lifetime and increasing operation cost, which is widely considered to be the bottleneck problem obstacle for PVDF membranes in water treatment. The critical surface tension (γc) of PVDF is 31 mN·m^−2^ which represents the strong hydrophobic nature of the PVDF membrane (Liu et al., 2011). Obviously, the hydrophobic property of PVDF membranes is one significant cause of membrane fouling and limited applications (Chang et al., 2008; Du et al., 2009; Yuliwati and Ismail, 2011). Therefore, many efforts have been committed to combining ZnO with PVDF membranes for antifouling enhancement.

Hong and He (2014) prepared PVDF–ZnO composite membranes by a blending process. All membranes exhibited typical asymmetric cross-section structures. It was found that addition of ZnO nanoparticles caused smaller water contact angles (enhanced hydrophilicity) and, therefore, can result in promoted antifouling ability. Moreover, the PVDF–ZnO composite membranes possessed looser sublayer structures, which is helpful to promote the filtration performance. It is well-known that membrane porosity is a positive correlation with exchange velocity of solvent/non-solvent. The hydrophilic ZnO nanoparticles can accelerate the importation of water to casting membrane because they have a higher affinity with water. Meanwhile, the solvents flow out of the casting membrane in a higher speed. In this way, the hydrophilic ZnO nanoparticles promoted the exchange velocity, therefore causing larger cavities, which promise the looser sublayer and higher membrane flux. Additionally, ZnO nanoparticles are good photochemical catalysts which can be used as a photocatalytic self-cleaning of the PVDF membranes. Hong and He (2014) studied the photocatalytic self-cleaning performance of PVDF–ZnO membranes. They found that the photocatalytic self-cleaning can supply a 93% washing efficiency to the PVDF membrane. The reason is that the ultraviolet can display an effective degradation to the adsorbed pollutants on the membrane due to the excellent photocatalytic property of ZnO nanoparticles.

Liang et al. (2012) mixed ZnO nanoparticles with PVDF to prepare PVDF–ZnO composite membranes to tackle irreversible membrane fouling. The experimental results indicated that water permeability of all four modified membranes was greatly improved because ZnO nanoparticles promoted the hydrophilicity of PVDF materials. But excess addition of ZnO nanoparticles can result in a slight permeability loss due to aggregations. After a simple physical cleaning, the permeation recovery rate of the modified membranes can reach to 100%, while that of the control PVDF membrane was only about 78%. This indicates that the PVDF–ZnO composite membranes have an obvious advantage in anti-irreversible fouling which is the intractable problem in membrane applications.

Zhang et al. (2014) prepared PVDF–ZnO composite membranes by internal and external modification methods. The static protein adsorption experiments revealed that control PVDF membrane had a bovine serum albumin (BSA) adsorption capacity of 0.128 mg·cm^−2^, whereas the PVDF–ZnO composite membrane showed the minimum adsorption capacity of 0.035 mg·cm^−2^. In addition, it was found that PVDF–ZnO composite membranes demonstrated higher adsorption property to heavy metal ions.

### Polyethersulfone Membrane Incorporated With Zinc Oxide

The presence of aromatic groups in PES determines the outstanding chemical and mechanical stability. Moreover, the PES possesses advantage in oxidative, thermal, and hydrolytic stability. For example, the high glass transition temperature of PES reaches to 230°C. Nevertheless, membrane fouling is the major problem for PES membranes as well as for other polymeric membranes. Therefore, many studies have been published to explore PES membrane modified with ZnO nanoparticles for antifouling purposes.

Zhao S. et al. (2015) fabricated PES–ZnO composite membranes and found that PES–ZnO composite membranes displayed a more porous membrane structure. The dynamic water contact angle showed that the PES–ZnO composite membranes presented better hydrophilicity than pristine PES membrane. The strengthened surface hydrophilicity was attributed to the addition of ZnO nanoparticles. Moreover, compared with pristine PES membranes, PES-ZnO membranes' thermal stability was improved.

Li et al. (2015) firstly synthesized 10-nm ZnO nanoparticles by a sol–gel method. After that, they introduced the ZnO nanoparticles into polymer membranes. The permeability was promoted from 46.4 to 365.8 L·m^−2^ h^−1^. To investigate the fouling behavior of the fabricated membranes, three model solutions were used as organic foulant mediums (Zhao L. H. et al., 2015). The flux loss rate and flux loss degree of PES–ZnO composite membranes were significantly reduced after the filtration of all the pollutant solutions. It is known that removal of BSA fouling from the membrane is more difficult. However, flux recovery rate of the optimal composite membrane reached to 80% by a simple physical wash, while the flux recovery rate for pristine membrane was only around 25%. Moreover, the PES–ZnO composite membranes possessed 83% flux recovery rate to the mixture solution of sodium alginate (SA), humic acid (HA), and BSA, which indicates that the PES–ZnO composite membranes can be a potential application in the real water treatments.

In our previous work (Shen et al., 2012), the experiments verified that the hydrophilicity of PES–ZnO membrane was considerably enhanced by the water contact angle reduction from 79.92° to 54.86°. The composite membrane exhibited high water flux (125.40 kg·m^−2^) which represented a promotion of 254% without sacrificing the membrane rejection. The control membrane suffered 27.7% flux decrease, however, the optimal PES–ZnO membrane with 0.3 g ZnO displayed only 7.8% flux decrease, which means that the antifouling ability was improved by the addition of ZnO nanoparticles.

In order to fix the ZnO nanoparticles on the membrane surface, Jo et al. (2016) firstly introduced NH_2_ into PES and then chemically assembled ZnO nanoparticles onto the membrane surface. The pristine PES membrane did not show obvious antibacterial ability, which was evidenced by the antibacterial activity rates against *Escherichia coli* and *Staphylococcus aureus* that were 0.21 and 0.20, respectively. When the ZnO was added, the antibacterial activity of composite membrane rapidly increased to 6.1 for both *E. coli* and *S. aureus*.

Balta et al. (2012) found that ZnO-modified membranes showed an overall improvement by ultralow concentration addition. In addition, they found that the ZnO nanoparticles caused a significant effect on the membrane substructure via affecting the phase inversion process. Ahmad et al. (2015) incorporated ZnO nanoparticles into PES to significantly improve the treatment performance for humic acid (HA) solution. They also found that all the composite membranes displayed a superiority to control membrane. For example, the composite membranes not only presented high water flux but also had higher rejection. Moreover, they exhibited promoted antifouling ability during the filtration process of HA solutions.

### Other Polymeric Membranes Incorporated With Zinc Oxide

Kim et al. (2018) developed the polyurethane (PU)–ZnO composite material *via* combined surface modification technologies. The composite membrane revealed significant photocatalytic/antimicrobial activity which was favorable to a potential application in organic pollutant degradation and wastewater purification. Since the compatibility of the organic–inorganic material is a general concern, Li et al. (2010) studied the mechanical properties of a novel chitosan (CS)–ZnO nanoparticle composite membrane prepared by a sol-casting method. The dry and wet samples were tested by tensile strength and elongation, respectively. It was interestingly found that the composite membranes possessed an enhanced mechanical property, which was attributed to intramolecular and intermolecular hydrogen bonds. After the introduction of ZnO, the intermolecular hydrogen bond between chitosan molecules was weakened; meanwhile, the hydrogen bond between CS and ZnO was enhanced. Nevertheless, excessive addition of ZnO can make the composite membranes brittle. Besides, the tests of *Bacillus subtilis, E. coli*, and *S. aureus* obviously presented that ZnO provided significant antibacterial ability.

Graphene oxide (GO), as one of the most popular materials (Dreyer et al., 2010; Chabot et al., 2014; Li et al., 2020), was combined with ZnO by Mahlangu et al. (2017). The GO–ZnO nanocomposites were impregnated in the membrane to increase the drug repellent properties by reducing the membrane–solute hydrophobic interaction. When the membrane and organic matters had the same type of charges, the repulsion at the interface played a role to promote the solute rejection. However, the organic matter with opposite zeta potential to the membrane was still adhered on the membrane surface, which could be caused by the concentration polarization. The composite membrane possessed enhanced hydrophilicity, permeability, and antifouling ability to the pristine membrane.

Pintilie et al. (2018) tried to compare the effects of ZnO and TiO_2_ on the polysulfone (PS) membranes. In the experiments, the composite membranes were prepared by blending PS with ZnO, TiO_2_ nanoparticles, and ZnO–TiO_2_ hybrid particles. They claimed that ZnO–TiO_2_ had a more efficient effect on PS membrane than that of ZnO and TiO_2_ nanoparticles. This was attributed to the synergetic effect of ZnO and TiO_2_ nanoparticles. By a detailed observing, it was found that ZnO nanoparticles played a comparable or better role in promotion of membrane performance than the widely used TiO_2_ nanoparticles. This study sufficiently verifies that the ZnO nanoparticle is a successful alternative to TiO_2_ nanoparticle in modifying polymeric membranes.

## Conclusion

In the last decade, the polymer–ZnO composite membranes have been significantly adopted by a large mass of literature reports. Therefore, the current review makes a summary of the latest findings in polymeric membranes incorporated with ZnO nanoparticles for membrane fouling mitigation. Firstly, the membrane fouling, ZnO nanoparticles, and modification technologies were introduced. After that, the review made intensive discussions on PVDF–ZnO composite membranes, PES-ZnO composite membranes, and other composite membranes incorporated with ZnO nanoparticles. These reports revealed that ZnO nanoparticles can endow polymeric membranes with significant hydrophilic, photocatalytic, and antimicrobial activities which were favorable to a potential application in wastewater treatments. For future development, the current review gives out several crucial topics: (a) overcoming aggregation and leaking of ZnO nanoparticles in the polymeric membrane is a key research topic in the future of ZnO nanoparticle-modified polymeric membranes; (b) antifouling mechanism research is another key topic since it can not only supply a sufficient explanation to the antifouling results but also direct the modification of membranes; (c) the long-term application research should be strengthened in view of the antifouling performance in previous studies that was primarily evaluated in short-term laboratory tests through the model solutions.

## Author Contributions

All authors listed have made a substantial, direct and intellectual contribution to the work, and approved it for publication.

### Conflict of Interest

The authors declare that the research was conducted in the absence of any commercial or financial relationships that could be construed as a potential conflict of interest.

## Abbreviations

PVDF: polyvinylidene fluoride
PES: polyethersulfone
PVC: polyvinyl chloride
PS: polysulfone
PE: polyethylene
MF: microfiltration
UF: ultrafiltration
NF: nanofiltration
RO: reverse osmosis
DFT: density functional theory
CFD: computational fluid dynamics
ANN: artificial neural network
ALD: atomic layer deposition
BSA: bovine serum albumin
SA: sodium alginate
HA: humic acid
PU: polyurethane
CS: chitosan
GO: graphene oxide
NOM: natural organic matter
XDLVO: Derjaguin–Landau–Verwey–Overbeek.
